# Glucose Metabolism is a Better Marker for Predicting Clinical Alzheimer’s Disease than Amyloid or Tau

**Published:** 2022

**Authors:** Tyler C. Hammond, Ai-Ling Lin

**Affiliations:** 1 Sanders-Brown Center on Aging, University of Kentucky, Lexington, KY, USA; 2 Department of Neuroscience, University of Kentucky, Lexington, KY, USA; 3 Department of Pharmacology and Nutritional Sciences, University of Kentucky, Lexington, KY, USA; 4 Department of Radiology and Biological Sciences, University of Missouri, Columbia, MO, USA

Alzheimer’s disease (AD) research has long been dominated with communications regarding the amyloid hypothesis and targeting amyloid clearance through pharmacological therapies from the brain [1]. Unfortunately, this research strategy has yielded only one new FDA-accelerated approved therapeutic for early AD, and its clinical benefit still needs to be verified [2]. It may be time to employ a new strategy in AD therapeutics research. Hammond et al. reported that diminished uptake of glucose in the brain is a better marker for classifying AD than beta-amyloid (Aβ) or phosphorylated tau deposition [3]. The National Institute on Aging and the Alzheimer’s Association published revised guidelines for the diagnosis of AD to include the measurement of amyloid (A), tau (T), and neurodegeneration (N), when diagnosing and treating AD [4]. It is highly relevant to AD therapeutic research whether amyloid, tau, and neurodegeneration contribute equally to the progression of AD at all phases of the disease or in a matter dependent on disease phase. To be able to successfully treat or prevent AD, there is a pressing need to identify precision biomarkers that are sensitive to disease progression and able to predict onset of cognitive impairment [5].

Hammond et al. used an advanced statistical learning machine learning method, random forest, on data provided by the Alzheimer’s Disease Neuroimaging Initiative (ADNI) to measure the ability of beta-amyloid measured by positron emission tomography (Aβ-PET), phosphorylated tau measured in the cerebral spinal fluid (CSF-pTau), fluorodeoxyglucose measured by positron emission tomography (FDG-PET) and structural imaging measured by magnetic resonance imaging (MRI) to classify AD diagnosis. Their results demonstrated that amyloid, tau, and neurodegeneration have a phase-dependent impact on the development of AD. Aβ and pTau are better predictors of the early dementia status that is often defined as mild cognitive impairment (MCI), and neurodegeneration, especially low glucose uptake, is a better predictor of later dementia status, or clinical AD. A similar pattern emerges when they correlate the biomarkers to performance on memory and executive functioning tests.

Amyloid may be an appropriate target for early treatment of AD, but glucose metabolism should be investigated as a target for treating AD in later disease [6]. Targeting glucose metabolism and insulin resistance could be an important step in overcoming mitochondrial dysfunction and cholesterol metabolism failures due to aging and other AD risk factors and in restoring cognitive resilience [7]. The repeated failures in AD clinical trials could potentially be due to the attempt to treat AD by eliminating Aβ; it is likely too late to treat Aβ in the late disease that is manifested by significant cognitive decline [8]. Therefore, an appropriate treatment course for AD may include a phase-structured approach where Aβ and tau are targeted early in disease course and brain metabolic restoration is targeted in late disease. Findings from the current work may shift the paradigm for future development of AD therapeutics.

Glucose hypometabolism plays a potentially very important role in the development of AD. Decreases in glucose uptake in the important areas of the brain can’t sustain the necessary support of neuronal activity and lead to reduced cognitive function [9–11]. Reduced glucose metabolism in the brain is also associated with insulin resistance, which has been associated with an exacerbation of Aβ deposition [10,12]. A key characteristic of AD includes impaired signaling of insulin in the brain [13]; because of this, some have referred to AD as type 3 diabetes due to the effects of insulin resistance on memory decline and impaired cognitive function [14,15]. In line with this characterization, type 2 diabetes mellitus, hyperlipidemia, and obesity all lead to an increased risk of AD development [10,16]. Conversely, normal brain glucose metabolism is highly associated with cognitive resilience and AD treatment efforts should include a preservation of normal brain glucose uptake. Indeed, in aged individuals who were cognitively unimpaired, glucose uptake in the bilateral anterior cingulate cortex and anterior temporal pole was shown to correlate highly with global cognition, despite the Aβ depositions that were present in these individuals along with their positive *APOE* ε4 status [17]; the findings indicate that preservation of normal cognitive performance can be achieved despite the hallmark phenotype and genotype of the disease. A different group reported that impaired glucose uptake can predict AD using deep learning methods an average of 75.8 months prior to its final diagnosis with 82% specificity and 100% sensitivity [18].

The maintenance of healthy blood glucose metabolism in the brain should be a priority focus of AD treatment as a strategy of preserving cognitive resilience and ameliorating disease progression. Some example therapeutics that focus on glucose metabolism include intranasal insulin and the ketogenic diet. The goal of intranasal insulin therapy is to provide insulin to the central nervous system rapidly via the olfactory and trigeminal pathways without adversely affecting systemic insulin levels; early results showed improvement of AD symptoms [19–21]. While results from the most recent clinical trial were muddled with complications of device delivery of the insulin, further studies need to test the efficacy of intranasal insulin [22]. The administration of the ketogenic diet provides an alternative fuel to the brain in the form of ketone bodies; this is especially useful when glucose metabolism has been altered as a result of insulin resistance [23–26]. The ketogenic diet has been shown to affect Aβ and Tau deposition and Aβ clearance in MCI and AD patients while also modifying the gut microbiome and short-chain fatty acid production [27,28]. The gut microbiome is also responsible for providing secondary bile acids for digestion; it is possible that modulation of the gut microbiome by the ketogenic diet can fix bile acid production problems that have been associated with AD [29,30]. Another dietary intervention that may have promise includes the use of prebiotics, which have been shown to balance systemic metabolism and reduce neuroinflammation in the presence of the *APOE* ε4 genotype [31].

In addition to glucose metabolism, there may be other metabolic processes that underly AD. We have new research demonstrating our use of ultrahigh performance liquid chromatography-tandem mass spectroscopy to perform a metabolomics analysis in the University of Kentucky-Alzheimer’s disease center brain bank on the dorsolateral and medial prefrontal cortex of 158 participants who were classified as AD, mixed dementia, or cognitively normal [32]. We performed various statistical analyses to determine how the metabolites differed in gray-enriched matter vs. white-enriched matter, AD vs. control, early stage vs. late stage, and *APOE ε3 vs ε4*. We also correlated metabolites with cognitive decline as measured by the MMSE. We found that white matter has increased lipids compared to gray matter, AD has increased metabolites related to phospholipid metabolism and decreased metabolites related to amino acid metabolism compared to controls, late ε4 has decreased metabolites that reduce atherosclerosis and decreased metabolites related to the krebs cycle and oxidative phosphorylation compared to early ε4, late 3 has increase metabolites related to oxidative DNA damage, inhibitory transmitters, and disruptions in neuronal membranes, and decreased metabolites related to acetylcholine synthesis compared to early ε3, ε4 at an early stages has increased metabolites related to poor kidney function and altered sterol function compared to ε3, and cognitive decline is associated with increased dipeptides and phospholipids.

Our results provide evidence that metabolism may be related to the disease course and progression of AD and that these metabolic shifts differ based on disease stage and APOE genotype [33]. This evidence contributes to a fundamental understanding of metabolism in AD for designing, testing, and developing precision medicine treatments for AD. New therapies should focus on treating the underlying metabolic challenges associated with AD. There are many therapeutics that may show clinical utility in AD, including a plant-based diet to combat the effects of atherosclerosis in ε4 patients [34], a Mediterranean diet to combat DNA damage in ε3 patients [35], or intranasal insulin or the ketogenic diet for modifying metabolism as a whole [36,37].

There also may be metabolic, immune, and neural associations of the gut microbiome with AD. Patients with AD and MCI have been shown to have an altered gut microbiome profile, with prominent decreases in Bacteroides, Lachnospira, and Ruminiclostridium_9 and increases in Prevotella [38]. Additionally, Escherichia is increased in AD and MCI and *Escherichia coli* fragments have been found to colocalize with Ab plaques [39]. Changes of the gut microbiome can lead to neuroinflammation in AD through an increase of phenylalanine and isoleucine, which help to stimulate pro-inflammatory T helper 1 (Th1) cells, leading to M1 microglia activation [40]. Trimethylamine N-oxide (TMAO), a small molecule produced by the metaorganismal metabolism of dietary choline, is also higher in individuals with MCI and AD dementia compared to cognitively-unimpaired individuals, and elevated CSF TMAO is associated with biomarkers of AD pathology (phosphorylated tau and phosphorylated tau/Aβ_42_) and neuronal degeneration (total tau and neurofilament light chain protein) [41]. Some fungi are also associated AD and MCI and specific diets can alter their balance with the bacteria present in the gut [42]. The administration of bifidobacteria in an AD mouse model has improved behavioral abnormalities and modulated gut dysbiosis [43].

In summary, treatments for AD that focus on solely on Ab may be too simplistic to treat the complexities of the disease. It appears that Aβ and tau drive early disease, but that neurodegeneration, especially in the form of low glucose metabolism, may exacerbate later forms of the disease. It is important that normalization of healthy metabolism in the brain be investigated as a treatment. Hammond et al. showed that amyloid and tau are better predictors of MCI and that low glucose uptake is a better predictor of AD. This may explain in part why so many clinical trials attempting to modify Ab have failed: the strategy of treating Ab is employed too late after a person has already progressed to late stage disease. Thinking in the field regarding AD progression and therapeutics should be altered to reflect these findings.
